# Physiological Association between Limb Ballistocardiogram and Arterial Blood Pressure Waveforms: A Mathematical Model-Based Analysis

**DOI:** 10.1038/s41598-019-41537-y

**Published:** 2019-03-26

**Authors:** Peyman Yousefian, Sungtae Shin, Azin Sadat Mousavi, Chang-Sei Kim, Barry Finegan, M. Sean McMurtry, Ramakrishna Mukkamala, Dae-Geun Jang, Uikun Kwon, Youn Ho Kim, Jin-Oh Hahn

**Affiliations:** 1 Department of Mechanical Engineering, University of Maryland, College Park, MD USA; 2 School of Mechanical Engineering, Chonnam National University, Gwangju, Korea; 3 Department of Anesthesiology and Pain Medicine, University of Alberta, Edmonton, AB Canada; 4 Department of Medicine, University of Alberta, Edmonton, AB Canada; 5 Department of Electrical and Computer Engineering, Michigan State University, East Lansing, MI USA; 6 Device & System Research Center, Samsung Advanced Institute of Technology, Suwon Gyeonggi, Korea

## Abstract

By virtue of its direct association with the cardiovascular (CV) functions and compatibility to unobtrusive measurement during daily activities, the limb ballistocardiogram (BCG) is receiving an increasing interest as a viable means for ultra-convenient CV health and disease monitoring. However, limited insights on its physical implications have hampered disciplined interpretation of the BCG and systematic development of the BCG-based approaches for CV health monitoring. In this study, a mathematical model that can predict the limb BCG in responses to the arterial blood pressure (BP) waves in the aorta was developed and experimentally validated. The validated mathematical model suggests that (i) the limb BCG waveform reveals the timings and amplitudes associated with the aortic BP waves; (ii) mechanical filtering exerted by the musculoskeletal properties of the body can obscure the manifestation of the arterial BP waves in the limb BCG; and (iii) the limb BCG exhibits meaningful morphological changes in response to the alterations in the CV risk predictors. The physical insights garnered by the analysis of the mathematical model may open up new opportunities toward next generation of the BCG-based CV healthcare techniques embedded with transparency, interpretability, and robustness against the external variability.

## Introduction

Cardiovascular disease (CVD) is a leading cause of mortality and morbidity that produces immense health and economic impacts in the United States and globally^1^. Considering its prevalence and implications on the quality of life and healthcare cost, one ideal solution to effective prevention and treatment of CVD is to enable ubiquitous surveillance and monitoring of CV risk predictors based on ultra-convenient techniques. However, the majority of state-of-the-art techniques for non-invasive measurement and assessment of CV risk predictors suffer from inconvenience. Indeed, techniques such as carotid-femoral tonometry for pulse wave velocity measurement^2–7^, flow-mediated dilatation for endothelial function assessment^8–11^, and ankle-brachial index for peripheral artery disease screening^12–14^ necessitate at least a subset of the following inconvenience and discomfort: trained operators, specialized costly equipment, access to privately sensitive body sites, and interventions.

The ballistocardiogram (BCG), defined as the body movement in response to the blood ejected by the heart, is increasingly receiving interest as an emerging modality equipped with the great potential to realize ultra-convenient CV health monitoring and assessment by virtue of its direct relationship to CV functions^15^ and its amenity to ultra-convenient measurement. Indeed, early investigations have demonstrated that the BCG may have clinical value due to the close association between its waveform morphology and various cardiac events^16–21^. In addition, rapid advances in the electronics and wearable technology opened up the possibility to ultra-conveniently measure the BCG during daily activities^22–30^. These unique advantages combined, recent applications of the BCG to CV health monitoring have reported success in estimating a range of CV parameters and risk predictors: heart rate^22,23^, pulse transit time and pulse wave velocity^24,31–34^, arterial blood pressure (BP)^28,30,32,33^, stroke volume and cardiac output^29,32^, and cardiac contractility^35–37^ to list a few.

Despite reasonable success and demonstrated promise, there are a few critical challenges common to most, if not all, prior endeavors on the BCG-based CV health monitoring. One salient challenge is that prior endeavors lack in rigor in terms of insights related to the physical meaning of the BCG. In the absence of established physical understanding of the BCG, most prior efforts have pursued brute-force data-driven approaches in which the association between a set of subjectively selected features in the BCG waveform versus the target CV parameters and risk predictors of interest was sought^27–30,37^. The other salient challenge is that the BCG waveform morphology is known to exhibit large variability with respect to the measurement instruments, postures, and locations^36,38,39^. These challenges altogether complicate the interpretation of the successful data-driven associations obtained in prior works, as well as hamper the seamless translation and generalization of the compelling findings obtained for the BCG pertaining to a specific instrument, posture, and location to other instruments, postures, and locations. It is contended that a viable solution to address these challenges is to drastically enhance the physiological understanding of the BCG, its association with the underlying CV physiology, and its variability with respect to the alterations in the instrument, posture, and location. Such physical insights, if established and properly integrated with the ongoing success of the data-driven BCG-based approaches to CV health monitoring, may open up new opportunities toward next generation of BCG-based CV healthcare techniques embedded with transparency, interpretability, and robustness against the external variability.

In our recent study, we elucidated based on a mathematical model-based analysis that the force exerted on the body due to the blood ejected by the heart (called the “force BCG”) results from the arterial BP gradients in the ascending and descending aorta^40^, indicating that the morphology of the BCG waveform has a close association with the underlying aortic BP waveforms. Then, in a series of subsequent work, we illustrated that such a physical understanding may provide valuable insights in the disciplined interpretation of the BCG in terms of CV parameters and risk predictors as well as in the systematic development of the BCG-based techniques for CV health monitoring^32,33^. However, the relationship between the force BCG and the BCG actually measured by various instruments at the limb locations still remains mysterious. Elucidating the physical mechanisms responsible for the relationship may pave the way toward understanding how the force BCG is transmitted to upper and lower limb locations through compliant joints and viscoelastic tissues to elicit the limb movement responses as well as interpreting the physiological association between the limb BCG versus the arterial BP waves, CV parameters, and CV risk predictors. Motivated by such a promise, the objective of this study was to conduct a rigorous mathematical model-based analysis of the association between the morphology of the arterial BP waves, force BCG, and the limb BCG. A mathematical model to predict the limb BCG responses to the arterial BP waves in the aorta was developed and experimentally validated. Then, the validated mathematical model was analyzed to discover the association between the arterial BP waves and the corresponding limb BCG waveforms as well as to predict the impact of changes in the CV risk predictors on the morphology of the limb BCG waveforms.

This paper is organized as follows. Section 2 describes the experimental data used in this study, as well as the mathematical model and the details of its calibration and analysis. Section 3 summarizes the results, which are discussed and interpreted to elucidate the physiological association between the arterial BP waves and the limb BCG waveforms in Section 4. Section 5 concludes the paper with suggested future work.

## Methods

In an attempt to establish the physiological association between the limb BCG and the underlying arterial BP, a mathematical model that relates the arterial BP waves to the limb BCG was conceived. The validity of the mathematical model was assessed in both qualitative and quantitative ways: (i) by investigating its efficacy in predicting morphologically correct limb BCG waveforms (qualitative), and (ii) by investigating its efficacy in predicting the absolute intervals and amplitudes associated with the experimentally observed limb BCG waves with minimal calibration (quantitative). Then, the mathematical model was simulated with the “representative” BP waves obtained from the experimental data to yield the limb BCG waveforms, which were analyzed together with the arterial BP waveforms to discover the association between the two. Details follow.

### Experimental Data

Experimental data from our prior work were used to assess the validity of the mathematical model. Given that the mathematical model would serve as the basis to establish the association between the limb BCG and the arterial BP waves in this work, the efficacy of the mathematical model to predict physiologically plausible limb BCG waveforms when the arterial BP waves are inputted was the primary concern. Data from two prior work were leveraged to assess the validity of the mathematical model: (i) arterial BP waves measured at the ascending aorta and femoral artery (Data 1; N = 20; age: 64 +/− 9 years; gender: 17 male and 3 female), and (ii) scale displacement BCG and wrist acceleration BCG along with non-invasive brachial BP (Data 2; N = 10; age: 24 +/− 2.3 years; gender: 4 male and 6 female; weight: 64 +/− 11 kg; height: 165 +/− 10 cm). Data 1 was collected from patients undergoing cardiac surgery with cardiopulmonary bypass under the approval of the University of Alberta Health Research Ethics Board and written informed consent. Its experimental protocol and setup are described in detail in our prior work^41,42^. Data 2 was collected from young healthy volunteers under the approval of the University of Maryland Institutional Review Board and written informed consent^30^. In each subject, the scale displacement BCG was measured using a custom-built weighing scale while the wrist acceleration BCG was measured using a custom-built wrist-worn accelerometer. The non-invasive brachial BP wave was measured using a commercial equipment (ccNexfin, Edwards Lifesciences, Irvine, CA, USA). The measurements were simultaneously taken while the subject was standing still on the weighing scale with their arms placed at the side and the movement minimized. Both Data 1 and Data 2 were collected in strict accordance with the relevant institutional guidelines and regulations.

It is acknowledged that the use of arterial BP and limb BCG data collected from separate studies to validate the mathematical model is not ideal. However, considering that the intended context of use of the mathematical model in this work is to predict physiologically realistic BCG waveforms rather than to precisely reproduce the experimentally observed BCG waveforms, the use of such data was regarded as acceptable.

Before its application to the mathematical model for analysis, the two data were standardized by scaling the arterial BP waves in Data 1 such that its group-average mean and diastolic levels were matched to the corresponding levels associated with Data 2.

### Mathematical Model

**Figure 1:**
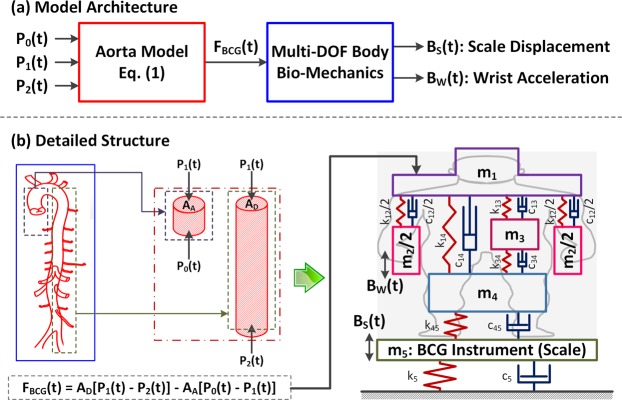
A mathematical model to predict ballistocardiogram (BCG) waveforms at the upper and lower limb locations in response to the heartbeat. (**a**) Model architecture: A mechanistic model that translates the heartbeat-induced aortic blood pressure (BP) waves to the force exerted on the body (called the force BCG) is integrated with a multi-degree-of-freedom (multi-DOF) mass-damper-spring model that represents the vibrational transmission in the body in the head-to-foot direction. (**b**) Detailed structure: The BP waves are inputted to the lumped-parameter mechanistic model of the aorta (1) to yield the force BCG. The force BCG subsequently excites the upper torso (m_1_) in the multi-DOF vibrational transmission model of the body to produce the corresponding movement (i.e., the BCG) of the upper limbs (m_2_) and lower limbs (m_4_). The lower limb BCG is measured as the resulting movement of the instrument (m_5_). Hence, the mathematical model predicts the scale displacement BCG as the displacement associated with m_5_, and the wrist acceleration BCG as the acceleration associated with m_2_.

A mathematical model to predict the BCG waveforms at the upper and lower limb locations in response to the heartbeat was conceived by integrating a mechanistic model translating the heartbeat-induced aortic BP waves to the force exerted on the body (called hereafter the “force BCG”) with a multi-degree-of-freedom (multi-DOF) mass-damper-spring model representing the vibrational transmission in the body in the head-to-foot direction (Fig. 1(a)). The former was adopted from our prior work^40^, which predicts the force BCG from three aortic BP waves: aortic inlet BP, aortic arch BP, and aortic outlet BP (Fig. 1(b)). In brief, the force BCG is the outcome of the interaction between the three aortic BP waves:

1 $${{\rm{F}}}_{{\rm{BCG}}}({\rm{t}})={{\rm{A}}}_{{\rm{D}}}[{{\rm{P}}}_{1}({\rm{t}})-{{\rm{P}}}_{2}({\rm{t}})]-{{\rm{A}}}_{{\rm{A}}}[{{\rm{P}}}_{0}({\rm{t}})-{{\rm{P}}}_{1}({\rm{t}})]$$

where F_BCG_ is the force BCG exerted on the body, P_0_, P_1_, and P_2_ are aortic inlet, arch, and outlet BP waves, respectively, and A_A_ and A_D_ are the ascending aortic and descending aortic areas. The latter was developed to fulfill two objectives: (i) to predict the vertical limb movements (i.e., the limb BCG) from the force BCG exerted on the upper torso; and (ii) to be minimally complex. An iterative trial and error process yielded a 4-DOF linear lumped parameter model consisting of four mass elements representing the upper torso (m_1_), upper limbs (m_2_), internal organs (m_3_), and lower limbs (m_4_), as well as the associated coupling elements to connect these masses (six dampers and six springs) (Fig. 1(b)). In this way, the essential behavior of the body in transmitting the force induced by the arterial BP waves to the upper (e.g., arm and wrist) and lower (e.g., leg and foot) limb sites may be captured.

To account for the fact that the measurement of the lower-limb BCG often requires a dedicated instrument (e.g., a weighing scale^26,35^), an additional mass-damper-spring dynamics (associated with the mass element m_5_ in Fig. 1(b)) was augmented to the above-mentioned mathematical model of the human body, so that the dynamic response characteristics of the instrument for the lower-limb BCG may also be accommodated in predicting the lower-limb BCG waveform (Fig. 1(b)).

Using the experimental data described in Section 2.1, the mathematical model was simulated as follows. The BP waves were inputted to the lumped-parameter mechanistic model of the aorta (1) to yield the force BCG. The force BCG subsequently excited the upper torso (m_1_) in the multi-DOF vibrational transmission model of the body to produce the corresponding movements (i.e., the BCG) of the upper limbs (m_2_) and lower limbs (m_4_). The lower limb BCG was measured as the resulting movement of the instrument (m_5_). Hence, the mathematical model predicts the scale displacement BCG as the displacement associated with m_5_, and the wrist acceleration BCG as the acceleration associated with m_2_.

### Parametric Sensitivity Analysis

To understand the overall variability of the limb BCG waveforms with respect to the variability in the bio-mechanical characteristics of the body as well as to determine the list of parameters in the mathematical model to calibrate using the experimental data, parametric sensitivity analysis was conducted as follows.

First, nominal parameter values for the mathematical model were determined. The parameters associated with the lumped-parameter mechanistic model of the aorta were adopted from the physically relevant values reported in the literature^40^. The parameters associated with the multi-DOF vibrational transmission model of the body were derived from the parameter values reported in a prior work on a comprehensive 16-DOF vibrational transmission model of human body^43^ via a standard model reduction procedure^44^. Specifically, the 16-DOF vibrational transmission model was reduced to the 4-DOF vibrational transmission model in Fig. 1 so that (i) m_1_ corresponds to the mass of the head and upper torso; (ii) m_2_ corresponds to the mass of the upper arms, elbows, forearms, and hands; (iii) m_3_ corresponds to the mass of the internal organs; and (iv) m_4_ corresponds to the mass of the thighs, shanks, and feet. The damping and stiffness parameters c_12_ and k_12_ associated with m_2_ as well as c_45_ and k_45_ associated with m_4_ were determined in such a way that the resulting fundamental resonance frequencies and amplitudes associated with m_2_ and m_4_ were matched to those associated with the corresponding subsystems in the 16-DOF vibrational transmission model^44^. On the other hand, the damping and stiffness parameters c_14_ and k_14_ connecting m_1_ and m_4_ as well as c_13_, c_34_, k_13_, and k_34_ connecting m_3_ to m_1_ and m_4_ were adopted directly from the respective values associated with the 16-DOF vibrational transmission model^43^.

Nominal parameter values associated with the instrument dynamics were assigned so that (i) m_5_ is the mass of the scale used to measure the lower-limb BCG in our prior work^30^; (ii) k_5_ and c_5_ yields the critically damped 1-DOF dynamics with the natural frequency reported in a prior study^26^. Second, the resulting 5-DOF vibrational transmission model was transformed into the transfer functions relating the force BCG to the scale displacement BCG and the wrist acceleration BCG:

2 $${{\rm{B}}}_{{\rm{S}}}({\rm{s}})={{\rm{H}}}_{{\rm{S}}}({\rm{s}}){{\rm{F}}}_{{\rm{BCG}}}({\rm{s}}),{{\rm{B}}}_{{\rm{W}}}({\rm{s}})={{\rm{H}}}_{{\rm{W}}}({\rm{s}}){{\rm{F}}}_{{\rm{BCG}}}({\rm{s}})$$

where B_s_(s) and B_w_(s) are the scale displacement and wrist acceleration BCG, and H_s_(s) and H_w_(s) are the associated transfer functions. Third, parametric sensitivity functions were analytically computed in the frequency domain as the partial derivatives of H_s_(s) and H_w_(s) with respect to the parameters therein:

3 $${{\rm{S}}}_{{\rm{S}},{\rm{\theta }}}({\rm{j}}{\rm{\omega }})=\frac{{{\rm{\theta }}}_{0}}{{{{\rm{H}}}_{{\rm{S}}}({\rm{j}}{\rm{\omega }})|}_{{\rm{\theta }}={{\rm{\theta }}}_{0}}}{\frac{\partial {{\rm{H}}}_{{\rm{S}}}({\rm{j}}{\rm{\omega }})}{\partial {\rm{\theta }}}|}_{{\rm{\theta }}={{\rm{\theta }}}_{0}},{{\rm{S}}}_{{\rm{W}},{\rm{\theta }}}({\rm{j}}{\rm{\omega }})=\frac{{{\rm{\theta }}}_{0}}{{{{\rm{H}}}_{{\rm{W}}}({\rm{j}}{\rm{\omega }})|}_{{\rm{\theta }}={{\rm{\theta }}}_{0}}}{\frac{\partial {{\rm{H}}}_{{\rm{W}}}({\rm{j}}{\rm{\omega }})}{\partial {\rm{\theta }}}|}_{{\rm{\theta }}={{\rm{\theta }}}_{0}}$$

where S_s,θ_(jω) and S_w,θ_(jω) denote the parametric sensitivity functions associated with H_s_(s) and H_w_(s), respectively, and ${\rm{\theta }}\in \{{\{{{\rm{m}}}_{{\rm{i}}}\}}_{{\rm{i}}=1}^{5},{{\rm{k}}}_{12},{{\rm{k}}}_{13},{{\rm{k}}}_{14},{{\rm{k}}}_{34},{{\rm{k}}}_{45},{{\rm{c}}}_{12},{{\rm{c}}}_{13},{{\rm{c}}}_{14},{{\rm{c}}}_{34},{{\rm{c}}}_{45},{{\rm{k}}}_{5},{{\rm{c}}}_{5}\}$ while θ_0_ is the nominal value of θ. Fourth, the sensitivity of the BCG morphology to the mass, damping, and stiffness parameters was analyzed in the frequency domain by way of the Bode magnitude plots of the parametric sensitivity functions. Finally, the results of this analytical parametric sensitivity analysis was confirmed by time-domain numerical simulation of the mathematical model, by examining and comparing the changes in the morphology of the scale displacement and wrist acceleration BCG waveforms entailed by the perturbations in the mass, damping, and stiffness parameters of the same percentage amount (+/−20%).

### Model Calibration

To evaluate the predictive capability of the mathematical model in Fig. 1 with respect to the experimental data described in Section 2.1, the mathematical model was calibrated to the experimental data. Considering that the primary role of the mathematical model is to provide the basis to elucidate the association between the limb BCG and the arterial BP waves, it is required that the mathematical model be able to predict typical limb BCG waveforms when typical arterial BP waveforms are inputted. Considering that a large portion of the nominal parameter values obtained for the mathematical model in Section 2.3 (e.g., the values of the mass parameters ${\{{{\rm{m}}}_{{\rm{i}}}\}}_{{\rm{i}}=1}^{5}$ and the stiffness parameters k_12_, k_13_, k_14_, k_34_, k_45_) may be physically appropriate to represent the body of an average subject according to the existing literature, the mathematical model was calibrated by optimizing a minimal set of parameters whose values are unknown and at the same time exert a large impact on the BCG morphology. Based on this rationale, all the mass and stiffness parameters with physical relevance (${\{{{\rm{m}}}_{{\rm{i}}}\}}_{{\rm{i}}=1}^{5}$ as well as k_12_, k_13_, k_14_, k_34_, k_45_) were fixed to the nominal values, whereas c_5_ and k_5_ (which are unknown) as well as high-sensitivity damping parameters (determined by the parametric sensitivity analysis) were calibrated to minimize the discrepancy between the experimental versus model-predicted BCG waveforms.

The calibration was performed specifically as follows. First, representative arterial BP waves were derived as the average of the arterial BP waveforms associated with all subjects in Data 1. Second, typical model-predicted BCG waveforms were derived using these arterial BP waveforms and the mathematical model in Fig. 1. The typical force BCG was computed as the output of the mechanistic model of the aorta (1) when the representative arterial BP waveforms were inputted. Then, the typical scale displacement and wrist acceleration BCG waveforms were computed by inputting the typical force BCG to the transfer functions H_s_(s) and H_w_(s). Third, the parameters c_5_ and k_5_ as well as the high-sensitivity damping parameters determined by the parametric sensitivity analysis were optimized in such a way that the difference between the experimental versus model-predicted BCG was minimized in terms of the amplitudes of the primary waves associated with the scale displacement (I, J, and K waves^40^) and wrist acceleration (J, K, and L waves^30^) BCG. For this purpose, representative wave amplitudes corresponding to the experimental BCG were derived as the average of the wave amplitudes associated with all subjects in Data 2. Then, the above-listed parameters were tuned by formulating and solving a numerical optimization problem to minimize the following penalty J using MATLAB and its Optimization Toolbox (MathWorks, Natick, MA):

4 $$\begin{array}{rcl}{\rm{J}} & = & {{\rm{J}}}_{{\rm{S}}}+{{\rm{J}}}_{{\rm{W}}}=[\mathop{\underbrace{{(\frac{{{\rm{I}}}_{{\rm{S}}}^{({\rm{M}})}-{{\rm{I}}}_{{\rm{S}}}^{({\rm{E}})}}{{{\rm{I}}}_{{\rm{S}}}^{({\rm{E}})}})}^{2}+{(\frac{{{\rm{J}}}_{{\rm{S}}}^{({\rm{M}})}-{{\rm{J}}}_{{\rm{S}}}^{({\rm{E}})}}{{{\rm{J}}}_{{\rm{S}}}^{({\rm{E}})}})}^{2}+{(\frac{{{\rm{K}}}_{{\rm{S}}}^{({\rm{M}})}-{{\rm{K}}}_{{\rm{S}}}^{({\rm{E}})}}{{{\rm{K}}}_{{\rm{S}}}^{({\rm{E}})}})}^{2}}}\limits_{{{\rm{J}}}_{{\rm{W}}}}]\\  &  & +\,[\mathop{\underbrace{{(\frac{{{\rm{J}}}_{{\rm{W}}}^{({\rm{M}})}-{{\rm{J}}}_{{\rm{W}}}^{({\rm{E}})}}{{{\rm{J}}}_{{\rm{W}}}^{({\rm{E}})}})}^{2}+{(\frac{{{\rm{K}}}_{{\rm{W}}}^{({\rm{M}})}-{{\rm{K}}}_{{\rm{W}}}^{({\rm{E}})}}{{{\rm{K}}}_{{\rm{W}}}^{({\rm{E}})}})}^{2}+{(\frac{{{\rm{L}}}_{{\rm{W}}}^{({\rm{M}})}-{{\rm{L}}}_{{\rm{W}}}^{({\rm{E}})}}{{{\rm{L}}}_{{\rm{W}}}^{({\rm{E}})}})}^{2}}}\limits_{{{\rm{J}}}_{{\rm{W}}}}]\end{array}$$

where I, J, K, and L are the amplitudes associated with the I, J, K, and L waves, the subscripts S and W denote the scale displacement and wrist acceleration, and the superscripts E and M denote experimental and model-predicted, respectively.

### Model Analysis

The mathematical model was subsequently used to assess the validity with respect to the experimental data, as well as to elucidate the physiological association between the limb BCG and the underlying arterial BP waves. Details follow.

The validity of the mathematical model was assessed with respect to the experimental data in two ways: pre-calibration qualitative assessment and post-calibration quantitative assessment. In the pre-calibration qualitative assessment, the mathematical model was evaluated for its ability to predict the presence of the primary waves in the scale displacement (I, J, and K waves) and wrist acceleration (J, K, and L waves) BCG. For the sake of this assessment, the un-calibrated mathematical model, equipped with the nominal parameter values obtained in Section 2.3, was excited with the arterial BP waves associated with all subjects in Data 1 to simulate the corresponding scale displacement and wrist acceleration BCG waveforms. Then, the number of subjects in which the presence of each of the primary waves was predicted in the simulated BCG waveforms was counted. In the post-calibration quantitative assessment, the mathematical model was evaluated for its ability to reproduce quantitatively correct BCG waveforms. For the sake of this assessment, the calibrated mathematical model was excited with the arterial BP waves associated with all subjects in Data 1 to simulate the corresponding scale displacement and wrist acceleration BCG waveforms. Then, the distributions of the primary wave-to-wave intervals (I-J and J-K intervals in the scale displacement BCG as well as J-K and K-L intervals in the wrist acceleration BCG) and wave-to-wave amplitudes (I-J and J-K amplitudes in the scale displacement BCG as well as J-K and K-L amplitudes in the wrist acceleration BCG) were computed (in terms of mean and standard error (SE)). These distributions were subsequently compared with the corresponding distributions obtained directly from the experimental BCG waveforms associated with all subjects in Data 2.

The physiological association between the limb BCG and the arterial BP waves was investigated in two ways. First, given that the primary constituents of the force BCG are the ascending aortic and descending aortic BP gradients^40^, the scale displacement and wrist acceleration BCG waveforms were decomposed into the components originating from the ascending and descending aortic BP gradients, and how each of these BP gradients are transformed into the BCG waveforms was investigated. The mathematical model is linear and the superposition principle applies. Hence, the decomposition reduces to simulating the mathematical model with the ascending and descending aortic BP gradients one at a time. This analysis was especially beneficial in scrutinizing the effect of individual arterial BP wave (P_0_, P_1_, and P_2_) on the BCG waveforms in that it enables how each arterial BP wave evolves into component waveform for the BCG; in contrast, direct analysis of the relationship between the combined ascending and descending aortic BP gradients and the resulting BCG waveforms may not yield much physiological insights due to the complex interaction among the three arterial BP waves. Second, the mathematical model was used to study the impact of the pulse wave velocity and pulse pressure amplification (which are well known CV risk predictors) on the morphology of the BCG waveforms. By using the representative arterial BP waves used in the calibration, the alterations in the pulse wave velocity and pulse pressure amplification were simulated by perturbing the time intervals and relative amplitudes between P_0_, P_1_, and P_2_. Specifically, an increase (or decrease) in the pulse wave velocity was realized by decreasing (or increasing) the time intervals between P_0_ and P_1_ as well as between P_0_ and P_2_ by the same percentage amount, while an increase (or decrease) in the pulse pressure amplification was realized by increasing (or decreasing) the pulse amplitude of P_2_ while maintaining the pulse amplitudes of P_0_ and P_1_. The representative arterial BP waves associated with the perturbations up to +/−20% in both pulse wave velocity and pulse pressure amplification were created. The nominal and perturbed representative arterial BP waves were inputted to the mathematical model to predict the resulting limb BCG waveforms. Then, the changes in the wave-to-wave intervals and amplitudes in the limb BCG in response to the alterations in the pulse wave velocity and pulse pressure amplification were investigated.

## Results

**Figure 2:**
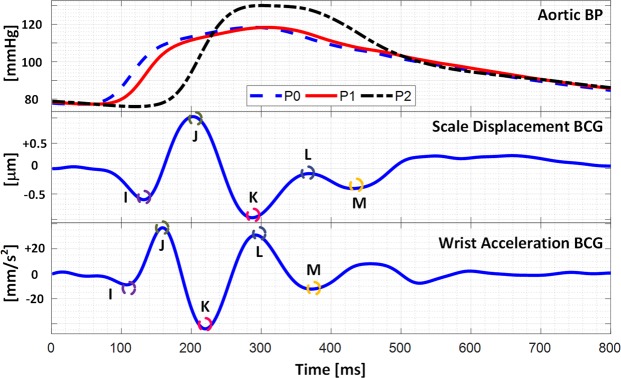
Representative arterial BP waves as well as pre-calibration model-predicted scale displacement and wrist acceleration BCG waveforms.

Figure 2 shows the representative (i.e., group-averaged) arterial BP waves as well as typical pre-calibration model-predicted scale displacement and wrist acceleration BCG waveforms. Overall, the mathematical model conceived in this study adequately predicted the overall morphology of the scale displacement and wrist acceleration BCG waveforms even without calibration to the experimental data. In particular, the presence of the primary waves (i.e., the I, J, and K waves in the scale displacement BCG as well as the J, K, and L waves in the wrist acceleration BCG) was observed in 95% of the subjects simulated with the experimental arterial BP waveforms (the K wave in the scale displacement BCG and the K and L waves in the wrist acceleration BCG were not clearly predicted in one subject). In addition, the BCG waveforms predicted by the 4-DOF mathematical model was almost identical to those predicted by the 16-DOF vibrational model^43^. Hence, it was concluded that the mathematical model used in this study is able to capture the essential characteristics associated with the transmission of the heartbeat-induced body movement throughout the body.

The parametric sensitivity analysis indicated that the most critical mass, damping, and stiffness parameters influencing the morphology of the scale displacement BCG turned out to be the arm mass (m_2_), spinal damping (c_14_), and scale stiffness (k_5_) parameters, while the most critical mass, damping, and stiffness parameters influencing the morphology of the wrist acceleration BCG turned out to be the arm mass (m_2_), spinal damping (c_14_), and arm stiffness (k_12_) parameters. Guided by these findings and motivated by the goal of predicting “typical” BCG waveforms, the mathematical model was calibrated by tuning c_14_ as well as c_5_ and k_5_ to minimize the discrepancy between the experimental versus model-predicted BCG wave amplitudes (see Section 2.4). Table 1 summarizes the parameter values in the mathematical model thus calibrated using the experimental arterial BP and limb BCG waveforms.

**Table 1:** Mathematical model parameter values calibrated using experimental arterial BP and limb BCG waveforms.

Mass [kg]		Damping [N1s/m]		Stiffness [kN/m]	
m_1_	9.0	c_12_	271	k_12_	40.7
m_2_	8.0	c_13_	53	k_13_	3.15
m_3_	23	c_14_	1056	k_14_	31.3
m_4_	25	c_34_	32	k_34_	2.28
m_5_	2.5	c_45_	1141	k_45_	425.3
		c_5_	722	k_5_	833.0

**Figure 3:**
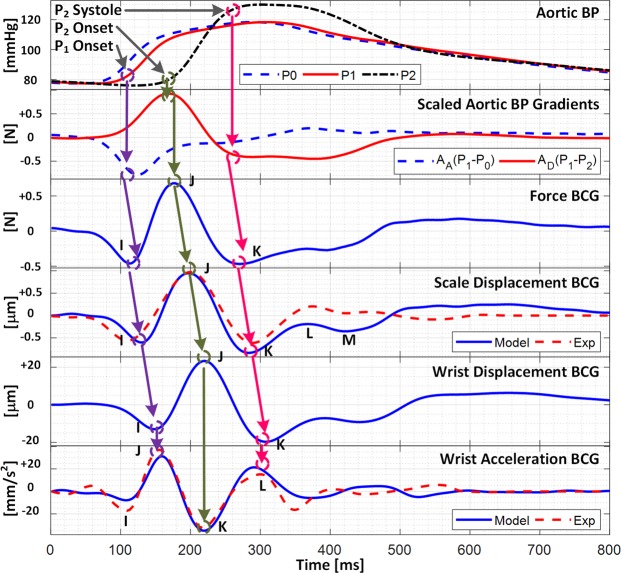
Force, scale displacement, wrist displacement, and wrist acceleration BCG waveforms predicted by calibrated mathematical model in conjunction with representative experimental scale displacement and wrist acceleration BCG waveforms.

Figure 3 shows the representative force, scale displacement, wrist displacement, and wrist acceleration BCG waveforms predicted by the calibrated mathematical model (by inputting the representative BP waveforms) in conjunction with the representative (i.e., group-averaged) experimental scale displacement and wrist acceleration BCG waveforms, while Table 2 summarizes the experimental and model-predicted wave-to-wave intervals and amplitudes in the scale displacement and wrist acceleration BCG waveforms. The mathematical model predicted the primary waves in the scale displacement (I, J, and K) and wrist acceleration (J, K, and L) BCG with small time interval (26% (I-J) and 5% (J-K) for scale displacement and 0% (J-K) and 15% (K-L) for wrist acceleration) and amplitude (6% (I-J) and 16% (J-K) for scale displacement and 3% (J-K) and 19% (K-L) for wrist acceleration) errors. On the other hand, its limited ability to reproduce the secondary H wave in the scale displacement BCG and the I wave in the wrist acceleration BCG is attributed to the fact that these waves are associated with the left ventricular activities^35^ while the mathematical model can only predict the BCG waves originating from the arterial BP gradients^40^.

**Table 2:** Wave-to-wave intervals and amplitudes in experimental and model-predicted ballistocardiogram (BCG) (mean +/− SE).

	Wave-to-Wave Intervals		Wave-to-Wave Amplitudes	
	I-J [ms]	J-K [ms]	I-J [µm]	J-K [µm]
**(a) Scale displacement BCG**				
Experiment (N = 10)	88 +/− 2	88 +/− 3	1.72 +/− 0.18	1.65 +/− 0.20
Model (N = 20)	70 +/− 3	92 +/− 6	1.63 +/− 0.20	1.97 +/− 0.15
Average Difference	18	4	0.09	0.32
	**J-K [ms]**	**K-L [ms]**	**J-K [mm/s** ^**2**^ **]**	**K-L [mm/s** ^**2**^ **]**
**(b) Wrist acceleration BCG**				
Experiment (N = 10)	62 +/− 3	80 +/− 3	73 +/− 7	52 +/− 4
Model (N = 20)	62 +/− 2	70 +/− 3	75 +/− 11	64 +/− 8
Average Difference	0	10	2	12

**Figure 4:**
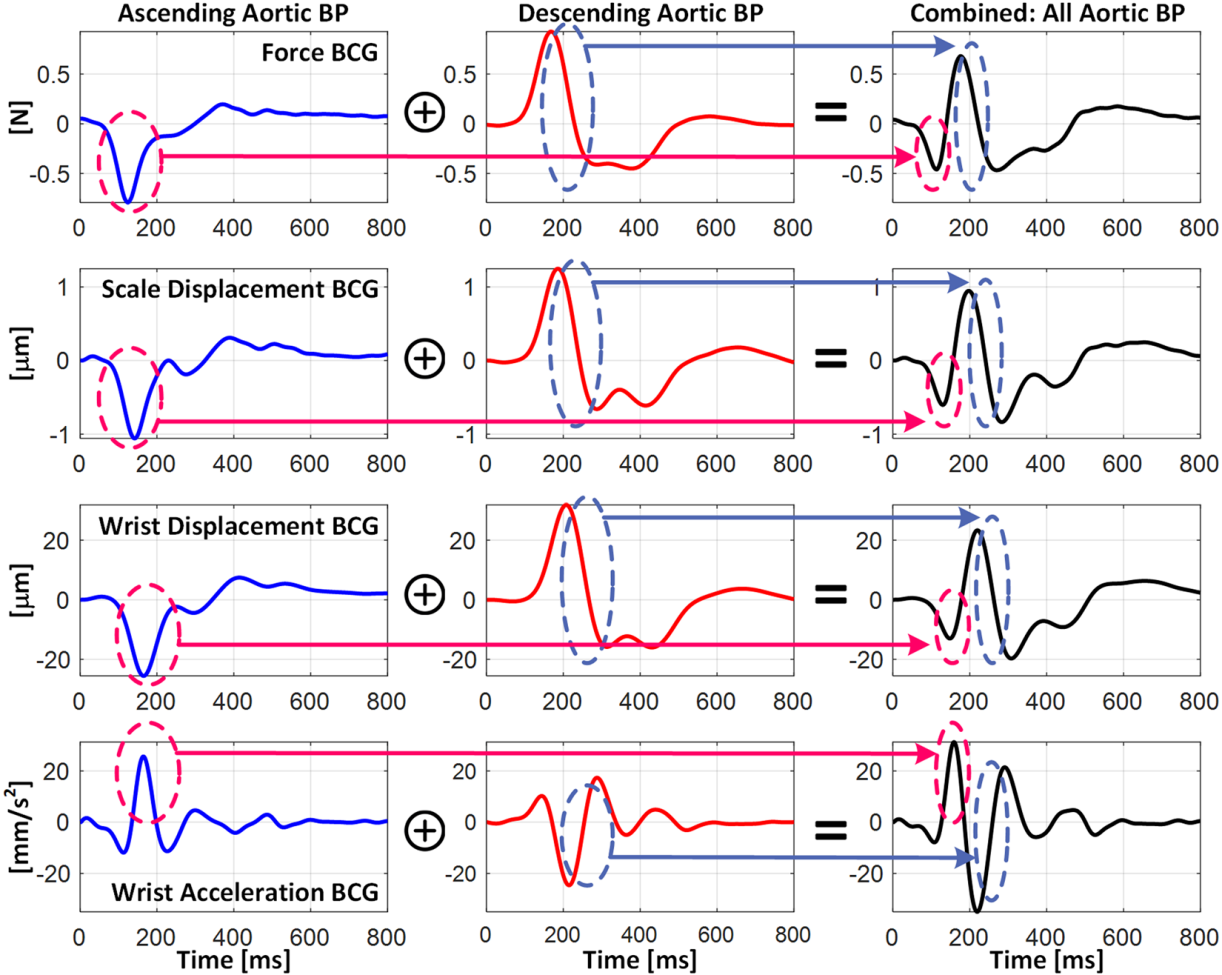
Decomposition of scale displacement, wrist displacement, and wrist acceleration BCG waveforms into components associated with ascending and descending aortic BP gradients.

Figure 4 shows the decomposition of the scale displacement, wrist displacement, and wrist acceleration BCG waveforms into the components associated with the ascending and descending aortic BP gradients. In both the scale and wrist displacement BCG, the falling limb of the I wave was primarily formed by the ascending aortic BP gradient, whereas the J-K down-stroke was predominantly formed by the descending aortic BP gradient. In the wrist acceleration BCG, accordingly, the J wave was mostly formed by the ascending aortic BP gradient, while the L wave was mostly formed by the descending aortic BP gradient. The K wave, on the contrary, was formed by both BP gradients, although the descending aortic BP gradient still had larger influence than its ascending counterpart. Yet all in all, the results shown in Fig. 4 suggest that all the I, J, and K waves in the displacement BCG as well as the J, K, and L waves in the acceleration BCG correspond to the same extrema in the underlying aortic BP gradients, illustrating that the pairs of (i) displacement I wave-acceleration J wave, (ii) displacement J wave-acceleration K wave, and (iii) displacement K wave-acceleration L wave are associated with the same physiological origins. These relationships are illustrated in Fig. 3 as well as summarized in Table 3.

**Table 3:** Relationships between arterial BP waves, arterial BP gradients, and scale displacement and wrist acceleration BCG waves.

Arterial BP	Arterial BP Gradients	Scale Displacement BCG	Wrist Acceleration BCG
P_1_ onset	Peak, P_0_-P_1_	I	J
P_2_ onset	Peak, P_1_-P_2_	J	K
P_2_ systole	Valley, P_1_-P_2_	K	L
P_1_ Amplitude	Positive Amplitude, P_1_-P_2_	J Amplitude	K Amplitude
P_2_ Amplitude	Peak-Peak Amplitude, P_1_-P_2_	J-K Amplitude	K-L Amplitude

**Figure 5:**
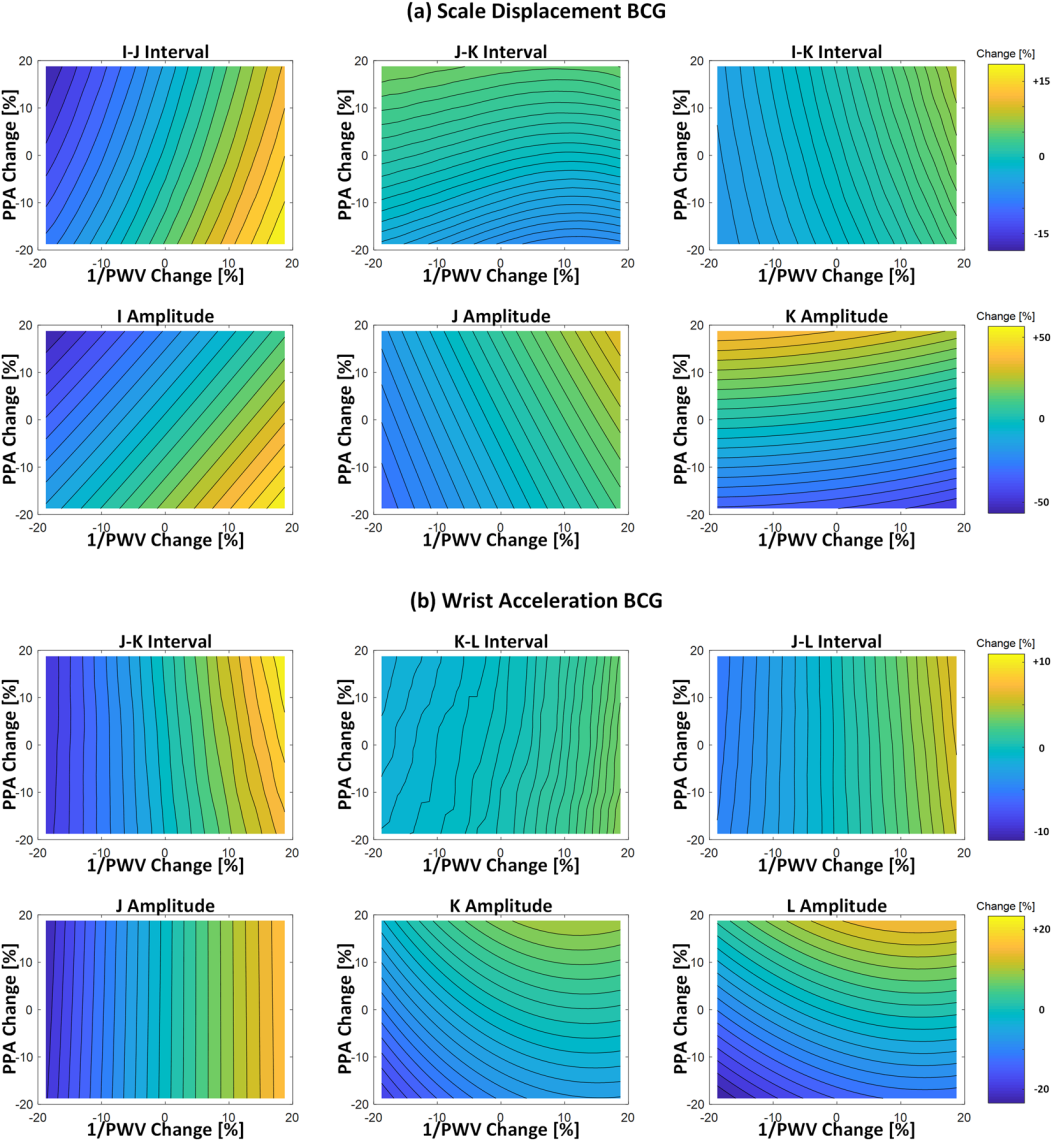
Relationship between the aortic pulse wave velocity (PWV) and pulse pressure amplification (PPA) versus the morphology of the limb BCG waveforms. (**a**) Scale displacement BCG. (**b**) Wrist acceleration BCG.

Figure 5 illustrates the relationship between the aortic pulse wave velocity and pulse pressure amplification versus the morphology of the limb BCG waveforms. Overall, the aortic pulse wave velocity was associated with both the wave-to-wave intervals and amplitudes in the limb BCG, whereas the aortic pulse pressure amplification was predominantly associated with the wave amplitudes in the limb BCG. In particular, an increase in the aortic pulse wave velocity yielded the corresponding decrease in (i) the I-J and I-K intervals in the scale displacement BCG, and accordingly, the J-K and J-L intervals in the wrist acceleration BCG; and (ii) the amplitudes of the I and J waves in the scale displacement BCG as well as the J wave amplitude in the wrist acceleration BCG. In addition, an increase in the aortic pulse pressure amplification yielded the corresponding increase in the J-K amplitude in the scale displacement BCG as well as the K-L amplitude in the wrist acceleration BCG.

## Discussion

### Mathematical Model: Validity and Implications

The calibrated mathematical model could faithfully reproduce the morphology of the scale displacement and wrist acceleration BCG waveforms (Fig. 3). In particular, the mathematical model predicted the presence of the I, J, and K waves in the scale displacement BCG as well as the J, K, and L waves in the wrist acceleration BCG. In addition, the agreement between the experimental and model-predicted wave-to-wave intervals and amplitudes were quite remarkable (Table 2). Considering that (i) the parameters in the mathematical model were only minimally calibrated (i.e., except for the scale-related parameters (c_5_ and k_5_), only one parameter (i.e., c_14_) was calibrated), (ii) they were fixed at constant values in predicting these waves associated with all subjects, and that (iii) the subjects associated with the BP waveforms used in the model prediction and those associated with the experimental BCG waveforms were largely different, the ability of the mathematical model to predict primary waves in the scale and wrist BCG with acceptable quantitative agreement with independent experimental data appears to strongly support the validity of the mathematical model in predicting the limb BCG waveforms. In fact, we speculate that a subset of the errors listed in Table 2 may in part be attributed to the discrepancy in the subject demographics associated with Data 1 and Data 2, and may be improved by reducing the gap associated with the subject demographics due to the following reasons. First, the model-predicted scale displacement BCG showed small I-J interval and amplitude as well as large J-K amplitude compared with its experimental counterpart, while the model-predicted wrist acceleration BCG showed large K-L amplitude compared with its experimental counterpart. Second, considering that the subjects in Data 1 may be associated with large pulse wave velocity and pulse pressure amplification compared with those in Data 2 (since the former are old and also subject to adverse CV state while the latter are young and healthy), the discrepancy in the CV state between these data may be removed by decreasing (i) the pulse wave velocity (e.g., by increasing the time interval between P_1_ and P_2_) and (ii) the PP amplification (e.g., by decreasing the amplitude of P_2_). According to Fig. 5, such alterations in pulse wave velocity and pulse pressure amplification will lead to the following changes in the model predictions: (i) an increase in the I-J interval of the scale displacement BCG, thereby improving the I-J interval accuracy in Table 2(a); (ii) an increase in the J wave amplitude and a decrease in the K wave amplitude in the scale displacement BCG, which will increase its I-J amplitude while maintain or decrease its J-K amplitude, thereby improving the I-J and J-K amplitude accuracy in Table 2(a); and (iii) a decrease in the K and L waves in the wrist acceleration BCG, which will largely decrease its K-L amplitude, thereby improving the K-L amplitude accuracy in Table 2(b).

The predicted BCG waveforms indicate that, as a first-order approximation, the I, J, K, and L waves in the wrist acceleration BCG may correspond to the H, I, J, and K waves in the scale and wrist displacement BCG for the following reasons. First, assuming that the body is rigid, all the body parts would undergo the same displacement, which would result in the identical scale and wrist displacement BCG waveforms. Second, considering that deriving wrist acceleration from wrist displacement involves two differentiations in time and also that differentiating twice in time leads to a phase lead of 180 degrees (along with frequency-dependent amplitude modulation), the gross morphology of the wrist acceleration BCG waveform may be derived by flipping (i.e., multiplying (−1) to) the wrist displacement BCG waveform.

Yet strictly, the body is not rigid; rather, it exhibits a complex multi-body dynamics nature comprising a number of mass, damping, and stiffness characteristics. In fact, the findings from the parametric sensitivity analysis suggest that the morphology of the limb BCG may be affected by the musculoskeletal properties of the subject, and the influence may not be negligible. In particular, both the scale displacement and wrist acceleration BCG were largely sensitive to the upper-limb properties among others. These musculoskeletal properties exert a mechanical filtering on the force BCG produced by the heartbeat, thereby altering the limb BCG waveforms (Figs 3 and 4). Therefore, the exact interpretation of the BCG to relate it to CV functions may require explicit account for the body dynamics.

### Association between Limb BCG and Arterial BP Waveforms

The mathematical model could now be exploited to elucidate the association between the limb BCG waves and arterial BP waves as follows.

First, the timings associated with the aortic BP waveforms may be indirectly deciphered from the limb BCG waveforms. More specifically, our prior work elucidated that the diastolic minima pertaining to the aortic inlet (P_0_) and outlet (P_2_) BP waves roughly correspond to the initiation of the I wave and the peak of the J wave in the force BCG^40^. Therefore, at least in an approximate sense, the I wave in the scale displacement BCG, the I wave in the wrist displacement BCG, and the J wave in the wrist acceleration BCG may indicate the diastolic minimum pertaining to the aortic inlet BP, and likewise, the J wave in the scale displacement BCG, the J wave in the wrist displacement BCG, and the K wave in the wrist acceleration BCG may indicate the diastolic minimum pertaining to the aortic outlet BP (Figs 3 and 4). Indeed, our prior experimental work suggests that the I wave in the scale displacement BCG can be used as the timing associated with the aortic inlet BP toward cuff-less BP monitoring^31–33^.

Second, the I wave amplitude in the scale displacement BCG and accordingly the J wave amplitude in the wrist acceleration BCG may represent the ascending aortic BP gradient. Indeed, Fig. 4 illustrates that the falling limb of the I wave in the scale displacement BCG as well as the rising limb of the J wave in the wrist acceleration BCG are determined primarily by the I wave in the force BCG (or equivalently, the ascending aortic BP gradient). Considering that the amplitude of the ascending aortic BP gradient is sensitive to the perturbations in the CV risk predictors of aortic pulse wave velocity and pulse pressure amplification, these waves may be analyzed to obtain meaningful insights on these CV risk predictors (Fig. 5).

Third, the J-K down-stroke in the scale displacement BCG and (accordingly) the K-L up-stroke in the wrist acceleration BCG may represent the descending aortic BP gradient. Indeed, Fig. 4 illustrates that the J-K down-stroke in the scale displacement BCG as well as the K-L up-stroke in the wrist acceleration BCG are determined primarily by the J-K down-stroke in the force BCG (or equivalently, the descending aortic BP gradient, in that the ascending aortic BP gradient is close to zero during this phase). Hence, together with the fact that the amplitude of the descending aortic BP gradient is sensitive to the distal pulse pressure^40^, these down-stroke/up-stroke portions may be analyzed to estimate distal pulse pressure.

Fourth, the J wave amplitude in the scale displacement BCG and accordingly (yet to a weaker extent) the K wave amplitude in the wrist acceleration BCG may represent the aortic pulse pressure. This speculation is plausible based on two observations: (i) the amplitude of the J wave in the force BCG may represent the aortic pulse pressure^40^; and (ii) the J wave in the scale displacement BCG and the K wave in the wrist acceleration BCG correspond approximately to the J wave in the force BCG. Hence, together with the surrogates of distal pulse pressure mentioned above (i.e., the J-K amplitude in the scale displacement BCG and the K-L amplitude in the wrist acceleration BCG), the limb BCG may provide a means to monitor another CV risk predictor of aortic pulse pressure amplification.

Finally, it must be noted that the promising potential of the limb BCG as surrogate measure of arterial BP hinges upon the significance of the mechanical filtering effect of the body. Indeed, the mechanical filtering has profound implications on the value of the limb BCG waveforms in probing arterial BP and CV functions. For example, the absolute timings associated with the arterial BP may not be robustly determined from the limb BCG compared to the force BCG, due to the non-negligible phase lag and morphological distortion imposed by the body’s mechanical filtering on the limb BCG waveforms.

Despite the confounding impact of body filtering, the mathematical model indicated that the time intervals between the primary waves in the limb BCG waveforms remained quite consistent. In particular, the I-J and J-K intervals associated with the force BCG (66 +/− 3 ms and 96 +/− 6 ms) and the scale displacement BCG (70 +/− 3 ms and 92 +/− 6 ms; Table 2) remained comparable. Further, these I-J and J-K intervals were also comparable to the J-K and (to a lesser extent) K-L intervals associated with the wrist acceleration BCG (62 +/− 2 ms and 70 +/− 3 ms; Table 2). In addition, the primary waves in both the scale (I, J, and K) and wrist (J, K, and L) BCG exhibited adequate degree of sensitivity in response to the changes in the arterial wave propagation characteristics (Fig. 5). Hence, the limb BCG may still possess value as surrogate measure of arterial BP and CV functions.

### Relationship between Aortic Pulse Wave Velocity and Pulse Pressure Amplification versus Limb BCG Morphology

By leveraging and compiling the mathematical model predictions illustrated in Figs 3–5, the following insights on the role of the aortic pulse wave velocity and pulse pressure amplification in shaping the limb BCG waveforms may be made.

First, the aortic pulse wave velocity influences the limb BCG morphology by altering the time intervals among the aortic BP waves (i.e., P_0_, P_1_, and P_2_ in Fig. 3). Regarding the wave-to-wave time intervals, the I-J and I-K intervals in the scale and wrist displacement BCG, and accordingly the J-K and J-L intervals in the wrist acceleration BCG as well, are inversely proportional to the aortic pulse wave velocity, because a decrease in the aortic pulse wave velocity results in the delay in the onset and peak timings of P_2_ (which delays the timings associated with the J and K waves in the scale displacement BCG and accordingly the K and L waves in the wrist acceleration BCG). Noting that the H wave in the scale displacement BCG roughly corresponds to the initiation of its I wave (and thus, the onset of P_0_^40^), the I-K and I-L intervals in the wrist acceleration BCG are also inversely proportional to the aortic pulse wave velocity. Regarding the wave amplitudes, the most salient influence of the aortic pulse wave velocity originates from the alteration of the separation between the ascending and descending aortic BP gradients. In particular, the amplitude of the I wave in the scale displacement BCG, and accordingly the amplitude of the J wave in the wrist acceleration BCG, are inversely proportional to the aortic pulse wave velocity, because a decrease in the aortic pulse wave velocity results in the greater separation between the two aortic BP gradients, weakening the mutual cancellation between them (in other words, the primary peaks associated with the ascending and descending aortic BP gradients are better preserved, leading to the scale displacement I wave and wrist acceleration J wave with higher amplitudes). Together with the observation that the amplitude sensitivity of all the other BCG waves to perturbation in the aortic pulse wave velocity was relatively small, the I-J amplitude in the scale displacement BCG, the I-J amplitude in the wrist displacement BCG, and the J-K amplitude in the wrist acceleration BCG (which may be proportional to the I-J amplitude in the scale displacement BCG) are also inversely proportional to the aortic pulse wave velocity.

Second, the aortic pulse pressure amplification influences the limb BCG morphology by altering the relative pulse amplitudes among the aortic BP waves. Its influence is primarily on the wave amplitudes (Fig. 5). Specifically, an increase in the aortic pulse pressure amplification (i.e., an increase in the pulse amplitude associated with P_2_ relative to P_0_ and P_1_) is associated with an increase in the J-K amplitude in the scale displacement BCG and accordingly the K-L amplitude in the wrist acceleration BCG, since the J-K down-stroke in the scale displacement BCG and the K-L up-stroke in the wrist acceleration BCG are determined by the level of pulse pressure associated with P_2_^40^.

Finally, two remarks are worth making. First, the absolute amplitude of the limb BCG waveform is directly proportional to the level of pulse pressure^40^. Hence, an overall increase in the level of pulse pressure (e.g., with aging) may increase the amplitude of the limb BCG waveform. Second, as a first-order approximation, the influence of the aortic pulse wave velocity and pulse pressure amplification on the limb BCG waveform morphology may appear de-coupled: (i) the wave-to-wave intervals are primarily affected by the aortic pulse wave velocity, and (ii) the wave amplitudes primarily altered by the aortic pulse wave velocity (scale displacement I-J and wrist acceleration J-K) and pulse pressure amplification (scale displacement J-K and wrist acceleration K-L) are distinct. However, in reality, both the aortic pulse wave velocity and pulse pressure amplification exert impacts on the limb BCG waveform morphology, and these influences can be quite convoluted (meaning that it may not be trivial to infer the alteration in the aortic pulse wave velocity and pulse pressure amplification from the rudimentary analysis of the changes in the limb BCG waveform morphology). Hence, the physiological insights obtained from the mathematical model analysis may need to be integrated with data-driven techniques (e.g., machine learning) to decipher CV states and functions from the limb BCG.

## Conclusion

The physiological association between the limb BCG waveforms and the arterial BP waveforms in the aorta was elucidated. It was demonstrated that arterial BP waves in the aorta may exert profound influences on the morphology of the limb BCG waveforms, and also that the influences are subject to complex interplay between the arterial BP waves. These findings suggest that certain characteristic features in the limb BCG waveforms may serve as viable surrogates of CV function, health, and potentially CVD. However, the convoluted and multi-faceted effects of the variability in the arterial BP waves on the limb BCG as well as the confounding impact of the bio-mechanical variability of the body may present challenges toward the development of novel techniques to decipher CV health and CVD from the limb BCG. Future effort must be invested to establish more rigorous physiological understanding of the limb BCG, examine the effect of CV pathophysiology on the morphology of the limb BCG waveforms, and investigate the opportunities to incorporate physiological insights derived from this study into CV health and CVD monitoring based on the limb BCG.
